# Ultrasound of ankles in the diagnosis of complications of chikungunya fever

**DOI:** 10.1590/0100-3984.2016.0221

**Published:** 2017

**Authors:** Roberto Mogami, João Luiz Pereira Vaz, Yêdda de Fátima Barcelos Chagas, Rodrigo Sperling Torezani, André de Almeida Vieira, Ana Célia Baptista Koifman, Yasmin Baptista Barbosa, Mirhelen Mendes de Abreu

**Affiliations:** 1 PhD, Adjunct Professor of Radiology at the School of Medical Sciences of the Universidade do Estado do Rio de Janeiro (UERJ), Rio de Janeiro, RJ, Brazil; 2 PhD, Adjunct Professor of Rheumatology at the Universidade Federal do Estado do Rio de Janeiro (Unirio), Rio de Janeiro, RJ, Brazil; 3 MD, Resident in Rheumatology at the Hospital Universitário Gafrée e Guinle (HUGG) da Universidade Federal do Estado do Rio de Janeiro (Unirio), Rio de Janeiro, RJ, Brazil; 4 MD, Resident in Radiology at the Hospital Universitário Pedro Ernesto (HUPE) da Universidade do Estado do Rio de Janeiro (UERJ), Rio de Janeiro, RJ, Brazil; 5 Undergraduate Medical Student at the Universidade do Estado do Rio de Janeiro (UERJ), Rio de Janeiro, RJ, Brazil; 6 PhD, Adjunct Professor of Rheumatology at the Universidade Federal do Rio de Janeiro (UFRJ), Rio de Janeiro, RJ, Brasil

**Keywords:** Chikungunya virus, Arboviruses, Ultrasonography, Synovitis, Tenosynovitis

## Abstract

**Objective:**

To describe the main ultrasound findings of chikungunya fever in the ankle.

**Materials and Methods:**

This was a cross-sectional observational study involving 52 patients referred to the Hospital Universitário Pedro Ernesto and presenting with clinical and biochemical evidence of chikungunya fever. The examinations were performed by a radiologist with more than 20 years of experience in ultrasound.

**Results:**

The predominant gender was female (in 88.5%), and the mean age was 58.4 years. The majority (61.5%) of the patients came from the northern part of the city of Rio de Janeiro, and 46.2% were using corticosteroids to treat inflammatory symptoms. The most common alterations observed by ultrasound were joint effusion (in 69.2%), tenosynovitis (in 59.6%), cellulitis (in 46.2%), Kager's fat pad thickening (in 29.9%), myositis (of the soleus or flexor hallucis longus muscle) (in 17.3%), retrocalcaneal bursitis (in 5.8%), tendon ruptures (in 3.8%), and increased vascular flow on power Doppler (in 3.8%).

**Conclusion:**

Signs of synovitis and tenosynovitis were the main ultrasound findings in a predominantly female population with a mean age of 58.4 years. Further studies are needed in order to define the role of ultrasound in the follow-up of such patients.

## Abstract (pt)

**Objetivo:**

Descrever os principais achados ultrassonográficos da febre chikungunya no tornozelo.

**Materiais e Métodos:**

Estudo transversal e observacional com 52 pacientes encaminhados ao Hospital Universitário Pedro Ernesto com quadros clínico e laboratorial compatíveis com febre chikungunya. Os exames foram realizados por um radiologista com mais de 20 anos de experiência no método.

**Resultados:**

Houve predomínio do sexo feminino (88,5%) e média de idade dos pacientes de 58,4 anos. A maioria dos doentes (61,5%) era proveniente da zona norte da cidade do Rio de Janeiro e fazia uso de esteroides (46,2%) para o tratamento dos sintomas inflamatórios. As alterações ultrassonográficas mais comuns foram: derrame articular (69,2%), tenossinovites (59,6%), celulite (46,2%), espessamento da gordura de Kager (29,9%), miosite (sóleo e/ou flexor longo do hálux) (17,3%), bursite retrocalcânea (5,8%), roturas tendíneas (3,8%) e hiperfluxo vascular pelo Doppler de amplitude (3,8%).

**Conclusão:**

Predominaram os sinais ultrassonográficos de sinovite e tenossinovite numa população majoritariamente do sexo feminino e com idade média de 58,4 anos. Sugere-se a realização de outros estudos para definição do papel da ultrassonografia no acompanhamento desses doentes.

## INTRODUCTION

Chikungunya fever is a disease caused by a virus of the family Togaviridae, genus *Alphavirus*, transmitted by the mosquitoes *Aedes aegypti* and *Aedes albopictus*^(1-4)^. Most infected patients become symptomatic, and the disease progresses through three phases: an acute phase, which can last up to 10 days and is characterized by fever, pain, periarticular edema, and, in many cases, rash^(2)^; a subacute phase, typically lasting from day 11 to month 3, in which rheumatic symptoms, characterized by synovitis (of the large and small joints) and tenosynovitis, predominate; and a chronic phase, beginning after the third month of evolution, in which there is persistence of inflammatory symptoms at some sites, myalgia, and peripheral nerve compressive syndromes. A small percentage of patients in the chronic phase develop profiles resembling that of rheumatoid arthritis, and the main finding on imaging studies is joint erosion^(1,5,6)^.

The chikungunya virus was initially isolated in Tanzania in 1952. After sporadic outbreaks of chikungunya fever in Africa and Asia throughout the 1960s and 1970s, the disease became statistically significant following an epidemic in Kenya in 2004 and especially after the epidemic of the Reunion Islands (in the Indian Ocean) in 2005. The first cases in the Western Hemisphere occurred in the Caribbean in 2013, and the virus spread to South American countries thereafter^(5,7-11)^.

In Brazil, autochthonous cases were initially identified in 2014 in the municipality of Oiapoque, located in the northern region of Brazil^(12)^. In 2015, there were 23,431 probable cases of chikungunya fever in Brazil, which rose to 236,287 cases in 2016 (a 1,008.4% increase), only up to the month of September. In 2016, the northeastern region accounted for 88.2% of these cases and the southeastern region for accounted for 8.0%. Of the cases of chikungunya fever in the southeastern region in 2016, the city of Rio de Janeiro accounted for 71.5%. The proportional increase in the number of cases in 2016 (up through the October-November period) in the city of Rio de Janeiro, in comparison with 2015, is even more absurd: 33,270%. That figure corresponds to 0.2% of the population of the city, according to the 2010 census. If we remember that in the Reunion Islands 30% of the population was affected by the virus, we can have an idea of the magnitude of the problem in Brazil^(13-15)^.

As a result of the outbreak in Rio de Janeiro, in the first half of 2016, emergency rooms in the city were crowded with patients complaining of fever and severe arthralgia. In a study published in a radiology journal, Mogami et al.^(8)^ was the first to mention the Rio de Janeiro outbreak and to describe the ultrasound alterations characteristic of chikungunya fever. The present study continues that line of research. The main objective was to describe the relevant changes that occur in the ankle as a result of infection with the chikungunya virus.

## MATERIALS AND METHODS

This was a cross-sectional observational study involving 52 patients referred to the Radiology Department of Hospital Universitário Pedro Ernesto, in the city of Rio de Janeiro, Brazil, between June and October of 2016. We included adult patients (≥ 18 years of age), of either gender, with history of sudden-onset fever, joint pain at multiple sites, with or without rash, and laboratory test results indicating IgM/IgG positivity for a diagnosis of chikungunya fever. Patients with rheumatic diseases such as rheumatoid arthritis, spondyloarthropathy, gout and septic arthritis were excluded, as were those with sequelae of fractures. The study was approved by the Research Ethics Committee of Hospital Universitário Pedro Ernesto (Protocol no. 58066716.8.0000.5259). All participating patients gave written informed consent.

The ankle ultrasound examinations were performed by a radiologist with more than 20 years of experience in the method. In addition to the routine investigation of the ankle, the same examiner evaluated the state of the calf muscles to identify any signs suggestive of myositis. A total of 104 examinations were performed with an ultrasound system (Aplio XG; Toshiba Medical Systems, Otawara, Japan), equipped with a multifrequency linear transducer (14–18 MHz in Bmode) and with power Doppler.

## RESULTS

Of the 52 patients in the sample, 6 (11.5%) were male and 46 (88.5%) were female. The mean age was 58.4 years.

The patients were stratified by their region of residence within the city, which was divided into four main parts: 32 (61.5%) resided in the suburban, northern part of the city; 10 (19.2%) resided in the western part; 9 (17, 3%) resided in the periphery (within the greater metropolitan region) of the city, and only 1 (1.9%) resided in the most exclusive, southern part of the city.

The most common symptoms, in descending order, were joint pain, in 52 patients (100%); fever, in 46 (88.5%); skin changes, in 37 (71.2%); pruritus, in 31 (59.6%); paresthesias, in 22 (42.3%); and lymph node enlargement, in 4 (7.7%).

At the time of the tests, 24 (46.2%) of the patients were using corticosteroids, 14 (26.9%) were taking no medication, 6 (11.5%) were using anti-inflammatory drugs, and 5 (9.6%) were using a combination of corticosteroids and immunosuppressants.

On average, the ultrasound examinations were performed 4.1 months after the onset of the disease, corresponding to the chronic phase.

**Figure 1:**
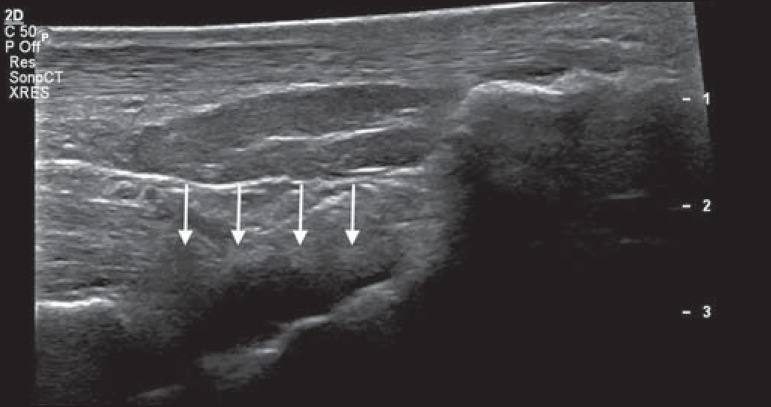
Longitudinal sagittal image of the tibiotarsal joint space, performed with a linear transducer at 14 MHz, showing hypoechoic areas (arrows), characteristic of effusion.

**Figure 2:**
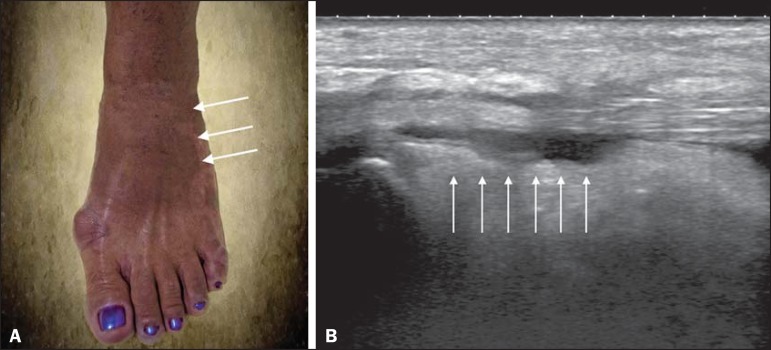
**A:** Photo of the dorsal region of the left foot, which shows anterolateral bulging (arrows). **B**: Corresponding to the alteration observed in the physical examination, ultrasound of the long extensor tendon of the fingers, performed with a linear transducer at 14 MHz and acquired in the sagittal plane, shows distention of the tendon sheath by an anechoic fluid collection (arrows), which is characteristic of tenosynovitis.

**Figure 3:**
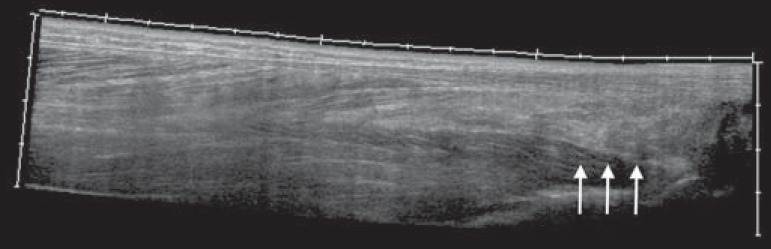
Sagittal ultrasound image of the posterior calf region, performed with a linear transducer at 14 MHz, showing thickening and increased echogenicity of Kager's fat pad (arrows).

**Figure 4:**
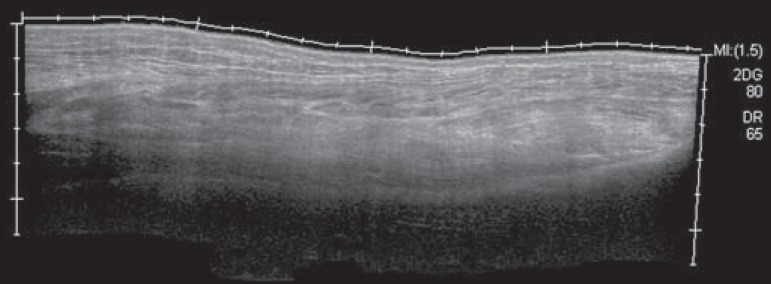
Sagittal panoramic view of the calf, performed by sweeping with a linear transducer at 14 MHz, showing diffuse hyperechogenicity of the soleus and flexor hallucis longus muscles, caused by myositis.

**Figure 5:**
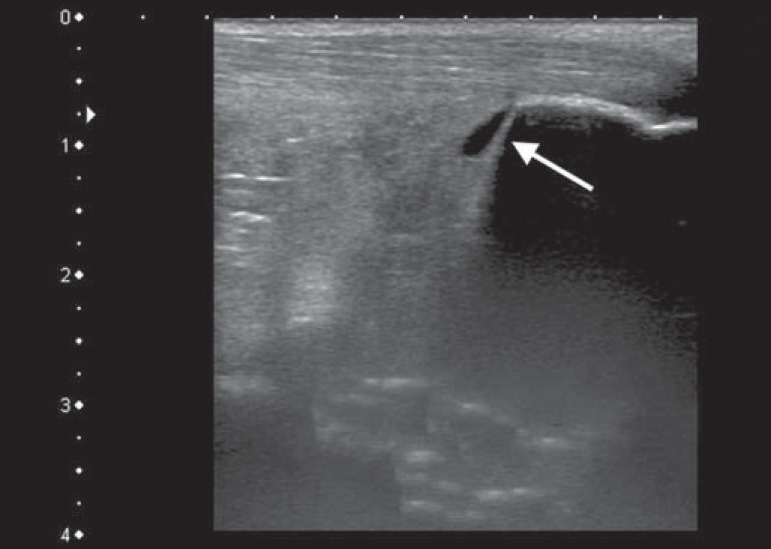
Sagittal image of the lower calf, performed with a linear transducer at 14 MHz, showing an anechoic fluid collection in the retrocalcaneal bursal projection (arrow), which is characteristic of bursitis.

**Figure 6:**
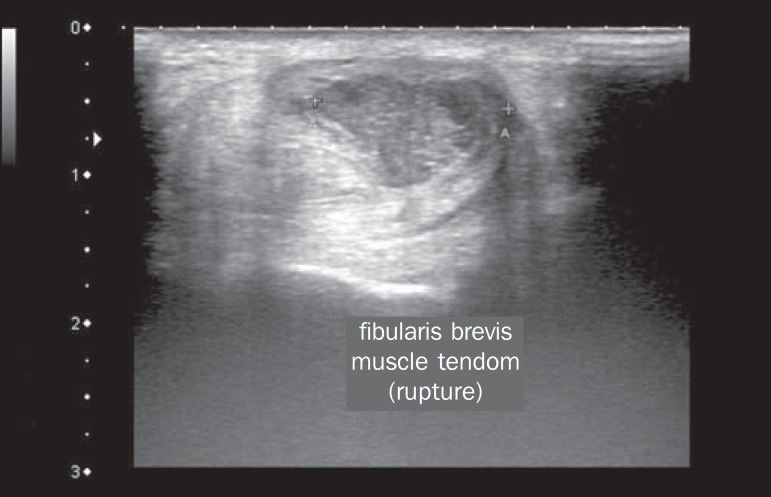
Cross-sectional image of the fibularis brevis muscle tendon, performed with a linear transducer at 14 MHz, showing an extensive hypoechoic area, indicative of a partial rupture, in the posterior region.

The most common ultrasound alterations in the ankles, in decreasing order of frequency, were joint effusion (Figure 1), in 36 (69.2%) of the patients; tenosynovitis (Figure 2), in 31 (59.6%); cellulitis, in 24 (46.2%), thickening of Kager's fat pad (Figure 3), in 14 (29.9%); myositis (of the soleus muscle, flexor hallucis longus muscle, or both) (Figure 4), in 9 (17.3%); retrocalcaneal bursitis (Figure 5), in 3 (5.8%); tendon ruptures (Figure 6), in 2 (3.8%), and increased vascular flow, as seen on power Doppler, in 2 (3.8%).

Fluid collections were located in the tibiotarsal joint space (Figure 1) in 27 (51.9%) patients and in the proximal intertarsal joint space in 23 (44.2%). Signs of tenosynovitis were found mainly in the posterior tibial sheath and fibular sheath-in 19 (36.5%) and 17 (32.7%) of the patients, respectively.

Pre-existing calcaneal tendon lesions were noted: calcifications were observed in 33 (63.5%) of the patients, and tendinopathy was observed in 6 (11.5%). In 13 patients (25%), there were no Achilles tendon lesions.

## DISCUSSION

The use of imaging examinations in the investigation of infections, especially those of regional incidence, have been the subject of various studies in the radiology literature of Brazil^(16-24)^. This paper presents the partial results of a larger, ongoing study at Rio de Janeiro State University, which is investigating musculoskeletal involvement of the upper and lower limbs in individuals with chikungunya fever. The studied population presented in the subacute or chronic phases of the disease, as can be seen by the mean time elapsed between the diagnosis and the performance of the examinations (4.1 months). There was a marked predominance of females, as well as of individuals in a higher age group (mean age, 58.4 years). These findings are similar to those of a study conducted on the island of Martinique^(25)^ and of the classic work on the chikungunya fever epidemic in the Reunion Islands^(5)^ but quite different from those of studies conducted in India^(26)^, Italy^(27)^, Sri Lanka^(3)^, and Colombia^(28)^. In the literature, it has been demonstrated that chikungunya fever patients over 35 years of age have an increased risk of developing chronic arthralgia^(29,30)^.

The fact that the majority of cases studied were in residents of the northern, suburban part of the city could be indicative of the vulnerability of that region to mosquito proliferation. However, it might also represent a geographical bias, due to the location of the hospital at which the study was located. However, data from the Rio de Janeiro Municipal Health Department show that during April of 2016, when the monthly number of reported cases of chikungunya fever was highest (5,109), the most affected neighborhoods were located in the northern and western parts of the city^(31)^.

All of the symptoms reported have been described in the literature as being associated with arbovirus infections, and chronic joint pain has been specifically associated with chikungunya^(8,26,32)^.

The heterogeneity of practices in relation to the treatment of patients reflects the lack of knowledge about the disease and the flow of patients referred from general or specialized outpatient clinics, as well as from emergency rooms in Rio de Janeiro.

Consistent with the literature, in our study, ankle involvement occurred symmetrically^(29,33,34)^. The two main alterations were effusion and tenosynovitis, as in other anatomical segments^(8,35,36)^. In the chronic phase of the disease, these collections are not very voluminous, although most, especially tenosynovitis, respond well to anti-inflammatory treatment. In the follow-up examinations, we observed cases in which there was regression of the inflammatory condition but persistence of residual asymmetric intra- or extra-articular foci, which merit additional study in order to identify any associated joint erosion.

Myositis, with or without cellulitis, was identified in 17.3% of the patients. The muscles most often affected in our sample were the soleus and flexor hallucis longus. Frequently, there was associated involvement of Kager's fat pad, which was hyperechoic and formed a continuous ultrasound aspect similar to that of myositis of the soleus muscle. The involvement is almost always symmetrical, and in the case of the lower limbs it is associated with complaints of fatigue and discomfort in the calf region. We believe that the bent position that gave rise to the name "chikungunya" in the Makonde dialect of Tanzania^(37)^ relates not only to ankle arthritis but also to generalized myositis of the sural musculature. In follow-up examinations, we found that myositis can persist for a long time, even when the patient responds well to steroid and immunosuppressive therapy. Because viral agents can cause idiopathic inflammatory myopathies, infection with the chikungunya virus might also trigger this type of condition in the long term^(38)^. Inflammatory involvement secondary to infection is a subject that has been discussed for some time, and the chikungunya virus has been associated with such involvement^(34)^.

In the present study, power Doppler showed inflammation in 3.8% of the ankles evaluated, as has been reported for the hands and wrists, albeit at an even lower frequency (Mogami, unpublished data). We believe that the greatest utility of this resource is in the follow-up of patients. Increased vascular flow in follow-up examinations might signal persistent inflammatory foci and alert the attending physician to the possibility of chronic inflammatory rheumatism^(25,28)^. That hypothesis should be tested in further studies.

One musculoskeletal comorbidity found in chikungunya fever patients was the involvement of the calcaneal tendon, either by enthesopathy (in 63.5%) or by tendinopathy (in 11.5%). In practice, this translates to amplification of symptoms existing prior to infection with the virus. Complaints of pain intensification are common during the chronic phase of the disease. Chen et al.^(10)^ stated that pre-existing degenerative musculoskeletal diseases could prolong the symptoms of arthralgia. Therefore, such comorbidities are of clinical relevance in the follow-up of these patients.

After the outbreak of chikungunya fever in 2016^(8,13,31)^, the expectation of the Brazilian health authorities is that the disease will return more forcefully in the summer of 2017. Despite the limitations of this study, the characterization and pioneering quantification of ankle lesions by ultrasound was an important means of highlighting the role that the method plays in the diagnosis of such complications. Additional studies are needed in order to evaluate the role that ultrasound plays in the monitoring individuals infected with the chikungunya virus and in the identification of cases that might progress to chronic inflammatory rheumatism or even secondary rheumatoid arthritis^(39)^. This need for follow-up studies of cases of chronic arthralgia with the potential for developing inflammatory arthritis has been cited by other authors^(10)^.

In summary, the 2015–2016 epidemic outbreak in Rio de Janeiro was characterized by the predominant involvement of older women who lived in the suburbs and by the lack of a clearly defined treatment for disease, most of them on steroids. The predominant findings in our series were effusion and tenosynovitis, mainly fibular and posterior tibial. The most common musculoskeletal comorbidity was involvement of the calcaneal tendon. Power Doppler was not very useful in the identification of areas with synovial inflammation.
